# Mass Extinction and the Disappearance of Unknown Mammal Species: Scenario and Perspectives of a Biodiversity Hotspot’s Hotspot

**DOI:** 10.1371/journal.pone.0150887

**Published:** 2016-05-18

**Authors:** Antonio Rossano Mendes Pontes, Antonio Carlos Mariz Beltrão, Iran Campello Normande, Alexandre de Jesus Rodrigues Malta, Antonio Paulo da Silva Júnior, André Maurício Melo Santos

**Affiliations:** 1 Universidade Federal de Pernambuco, Centro de Ciências Biológicas, Departamento de Zoologia, R. Prof. Moraes Rego, 1235, Cidade Universitária, Recife, Pernambuco—PE, Brazil; 2 Instituto Chico Mendes de Conservação da Biodiversidade—ICMBio, Estrada do Forte Orange, S/N, Caixa-postal: 01, Itamaraca, Pernambuco—PE, Brazil; 3 Universidade Federal de Pernambuco, Centro de Ciências Biológicas, Departamento de Zoologia, R. Prof. Moraes Rego, 1235, Cidade Universitária, Recife, Pernambuco—PE, Brazil; 4 Instituto Tecnológico do Estado de Pernambuco–ITEP, Avenida Professor Luiz Freire, 500—Cidade Universitária, Recife, Pernambuco—PE, Brazil; 5 Universidade Federal de Pernambuco, Núcleo de Biologia–Centro Acadêmico de Vitória, UFPE. Rua do Alto do Reservatório, S/N, Bela Vista, Vitória de Santo Antão, PE, Brazil; Centre for Cellular and Molecular Biology, INDIA

## Abstract

We aimed to determine the conservation status of medium- and large-sized mammals and evaluate the impact of 500 years of forest fragmentation on this group of animals in the Pernambuco Endemism Center, in the biogeographical zone of the Atlantic forest north of the São Francisco River in northeastern Brazil. Line transect surveys were performed in 21 forest fragments, resulting in a checklist of the mammals of the entire Pernambuco Endemism Center area. We ran a generalized linear model (Factorial ANCOVA) to analyze to what extent the vegetation type, fragment area, isolation, sampling effort (as total kilometers walked), or higher-order interactions predicted (a) richness and (b) sighting rates. To determine if the distribution of the species within the forest fragments exhibited a nested pattern, we used the NODF metric. Subsequently, we performed a Binomial Logistic Regression to predict the probability of encountering each species according to fragment size. Out of 38 medium- and large-sized mammal species formerly occurring in the study area, only 53.8% (n = 21) were sighted. No fragment hosted the entire remaining mammal community, and only four species (19%) occurred in very small fragments (73.3% of the remaining forest fragments, with a mean size of 2.8 ha). The mammalian community was highly simplified, with all large mammals being regionally extinct. Neither the species richness nor sighting rate was controlled by the vegetation type, the area of the forest fragments, isolation or any higher-order interaction. Although a highly significant nested subset pattern was detected, it was not related to the ranking of the area of forest fragments or isolation. The probability of the occurrence of a mammal species in a given forest patch varied unpredictably, and the probability of detecting larger species was even observed to decrease with increasing patch size. In an ongoing process of mass extinction, half of the studied mammals have gone extinct. The remaining medium-sized mammal community is highly simplified and homogenized. The persistence of these species in a forest patch is determined by their ability to adapt to a novel simplified diet, the efficient use of the surrounding matrix without being engulfed by the sink effect, and escaping hunting. Our results suggest that the 21^st^ century medium-sized mammalian fauna of this region will comprise only four species unless strict conservation measures are implemented immediately and every forest fragment is effectively protected.

## Introduction

The biodiversity crisis that threatens the planet can be considered the sixth major extinction event [1], and tropical forest deforestation is causing extinctions at unprecedented rates [2,3]. The biota of the future, or the subset of species that will remain after extinction-debts have played out, therefore, has already established itself in several areas of the world [4,5], presenting considerably simplified communities whose future is not assured.

Mammal species respond in different ways to fragmentation. Generalists are favored by the absence of larger predators and by their ability to exploit alternative resources [6,7,8,9,10]. In contrast, large predators, which require extensive home ranges, and have fewer individuals, are the first to disappear [11,12,13,14]. Additionally, fragmentation leads to the imminent risk of the sink effect [15], in which individuals try to cross the open matrix in search of suitable habitats, and are killed, which drives the remaining species to extinction. The number of species will therefore decline, reaching a new, less diverse state ([16,17]).

The Brazilian Atlantic forest is considered one of the most important biodiversity hotspots on the planet [18]. Within this forest, one of the important biogeographical regions is the Pernambuco Endemism Center (herein, CEPE). Located in northeastern Brazil north of the São Francisco river [19,20,21,22], the CEPE hosts a high number of endemic species, suggesting that it is a hotspot within a hotspot, i.e., the Pernambuco *refugium* [23,24].

Unfortunately, the CEPE has lost 94.4% of its forest cover [25,26] (this study), no longer has a large continuous forest, and most of its fragments are smaller than 10 ha [27], isolated in a non-forested matrix and exposed to severe human pressure [27,28,29]. A scenario inadequate to support the current mammalian fauna [30,7].

The CEPE biota have a comparatively longer history of human exploitation [7,31], which began earlier than the colonization process, as stated by Gandavo in 1575 and by Salvador in 1627, but it appears to have been intensified by the arrival of the first colonizers who were recorded exchanging goods for animals [32,33]. This long-term exploitation has resulted in a mass extinction without precedent in modern history [3,14,34,35], and the “living dead” species [15] that have persisted have suffered dramatic changes in their ecology and behavior [27,29,30,36,37,38].

As shown by Lees and Pimm [39] species are being lost in the Atlantic forest of northeastern Brazil soon after they are described, and in some cases, even before they were described, being known only from paintings from the first colonizers or oral testimonies. As they highlight, these species are presumed to go extinct quickly, unless extraordinary efforts are made to save them.

In order to confirm the occurrence of those mammals referred in the old literature as once occurring in the CEPE we decided to build a checklist of the medium- and large-sized mammals. Thus, between 2000 and 2008 we carried out a thorough review of the literature on the occurrence of these mammals in the region, using sources reaching back to the start of the colonization process in the 1500s. This provided unprecedented access to the first and only records of many medium- and large-sized mammals from the CEPE, some of which are currently extinct, and some others that have never been seen by contemporary scientists [32,33,40,41,42,43,44].

We also thoroughly surveyed 21 forest fragments from sites widely distributed within the highly fragmented landscape of the CEPE. These covered a range of sizes from 4 to 3,478.3 ha (the latter being the largest of the CEPE), and determined mammal species richness and abundance in each [8,10,14,35,45,46,47]. During these surveys we discovered the dwarf porcupine, *Coendou speratus* [48].

We found that the occurrence of some of the mammals referred in the old, previously unknown literature from the 16^th^ and 17^th^ century, and still considered widespread in the CEPE, have, in fact, not been validated by contemporary scientists (e.g. spotted paca, *Cuniculus paca*); were recognized as occurring in the CEPE only in the turn of the 21st century (e.g. tayra, *Eira barbara*), when they had already gone regionally extinct (e.g. bush dog, *Speothos venaticus*), or were never seen, such as a rufous long-tailed spider monkey (*Ateles* sp.) referred by Barlaeus in 1647 [42], a total novel contribution of this study to contemporary science.

Additionally, no fragment held the entire mammal community expected, most fragments had less than half of the mammals and no large mammals were found, namely, deer (*Mazama* spp.), white-lipped peccary (*Tayassu pecari*), jaguar (*Panthera onca)*, puma (*Puma concolor)*, Brazilian tapir (*Tapirus terrestris)*, and giant anteater, (*Myrmecophaga tridactyla*), revealing an ongoing unprecedented mass extinction in the CEPE [8,10,14, 35,45,46,47]. Our novel, unprecedented findings [35] were recently confirmed by Canale et al. [49], who interviewed local residents living in the vicinity of forest fragments of the nearby Bahia Endemism Center (herein, CEBA), another important hotspot within the Atlantic forest of northeastern Brazil. Again, interviewees had never seen jaguar (*Panthera onca*), giant anteater (*Myrmecophaga tridactyla*), white-lipped peccary (*Tayassu pecari*), and Brazilian tapir (*Tapirus terrestris*), reinforcing our unprecedented findings.

Thus, in order to test the hypothesis that it was the case of the entire remaining ~50 km^2^ of this hotspot´s hotspot, we compiled the two decades of systematic surveys that we had carried out in the various different landscapes of the CEPE [35], and aimed to answer the following questions: (1) Is the current number of species of medium- and large-sized mammals in the CEPE significantly reduced in comparison with that recorded by the first colonizers?; (2) To what extent do the vegetation type, fragment area, fragment isolation or any interactions among these factors predict the species richness and abundance of medium- and large-sized mammals in this severely fragmented area?; (3) Are the medium- and large-sized mammal species assemblages subsets of successively larger ones as a consequence of the ongoing selective extinction and isolation process [50,51]?; (4) How does each mammal species respond to variations in fragment size in terms of its probability of encounter?

## Materials and Methods

### Study area

This study was part of a larger research project, entitled: Biological Diversity and Conservation of the Atlantic Forest North of the São Francisco River, financed by the Probiota program (PROBIO), of the National Research Council (CNPq), of the Ministry of Environment (MMA), with which the project coordinator signed the agreement that allowed us to carry out the surveys. No licences were required to this specific study, or subproject, since we only observed the animals along pre-established trails and did not influence or interact with them in any invasive way (e.g. trapping, collecting, experimental manipulation, or sacrificing). Thus, we were allowed to carry out our observational field studies in all the areas, independently from its status, as part of the larger research team.

**Fig 1:**
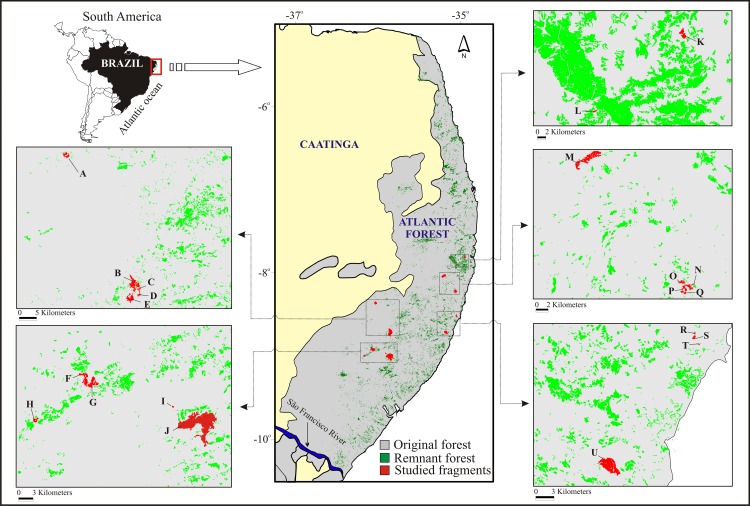
The study area in the Atlantic forest of northeastern Brazil, specifically, the Pernambuco Endemism Center–CEPE, showing its current and former forested area and the studied forest fragments (due to some fragments being too small, they do not appear individually) (Spatial database provided by Fundanção SOS Mata Atlântica / INPE, under a CC BY license). A—Brejo dos Cavalos (08°22'48"S; 36°02'24"W); B—Quengo (08°43'04"S; 35°50'27"W); C—Espelho (08°43'12"S; 35°50'40"W); D—Ageró (08°44'16"S; 35°50'33"W); E—Fervedouro (08°45'08"S; 35°51'36"W); F—Capoeirão (08°55'1"S; 36°04'17"W); G—Cachoeira (08°56'44"S; 36°03'36"W); H—Bom Jesus (09°01'16"S; 36°10'32"W); I—Aquidabã (08°58'53"S; 35°54'25"W); J—Coimbra (08°59'42"W; 35°50'27‴S); K—Charles Darwin (07°48'56"S; 34°57'11"W); L—Vale das Águas (7°54'49"S; 35°3'25,4"W); M—Tapacurá (08°03'00"S; 34°55'00"W); N—Café (08°14'12"S; 35°02'60"W); O—São Braz (08°13'28"S; 35°04'03"W); P—Cuxiu (08°13'38"S; 35°03'45"W); Q—Xangô (08°14'23"S; 35°03'56"W); R—Mingú (08°31'02"S; 35°03'15"W); S—Gengibre (08°31'33"S; 35°03'16"W); T—Bulandi (08°32'10"S; 35°02'50"W); U—Saltinho (08°45'00"S; 35°10'00"W).

The study was carried out between 2000 and 2008 in 21 forest fragments within the CEPE (Fig 1). The fragments were classified as very small (≤ 10 ha), small (10.1–100 ha), medium-sized (100.1–1,000 ha), or large (>1,000 ha; the largest is only 3,478.3 ha). The forest fragments were located in four of the best-protected forest archipelagos of the CEPE: the Gurjaú Ecological Reserve (1,077.10 ha), Usina Frei Caneca Mill (630.42 ha), Usina Serra Grande Mill (8,000 ha), and Usina Salgado Mill (1,000 ha) (Fig 1). We also studied three other officially protected areas: the Brejo dos Cavalos Ecological Park (354 ha), Tapacurá Ecological Station (382 ha), and Saltinho Biological Reserve (548 ha).

Additionally, two non-protected urban forest fragments in the cities of Camaragibe (8 ha) and Igarassu (60 ha) were included in the analyses (Fig 1). Finally, we opportunistically recorded all sightings in the surrounding matrix or open areas, which were used in producing the checklist of occurring species.

The investigated forest fragments measured from a minimum of 4 to a maximum of 3,478.3 ha. Following the classification of Oliveira & Fontes [52], these occurred in the following habitat types: lowland evergreen forest (n = 11), submontane semi-deciduous forest (n = 7), submontane evergreen forest (n = 2), or low submontane semi-deciduous forest (n = 1) (Fig 1). There is no continuous forest or control fragment, and all of the fragments have experienced some degree of human interference, such as hunting, the presence of feral dogs, forest clearing, selective cutting, or intentional fires, in addition to being bisected by roads used by people and domestic animals.

We calculated the total number of forest fragments for each size class in the entire remaining CEPE using ArcGis 9.2 software from supervised classifications of LANDSAT 3 and 4 (dated between 1973 and 1979) and 5 and 7 (dated between 2001 and 2007) satellite images (Instituto Nacional de Pesquisas Espaciais—INPE), selected for least cloud cover. Lastly, we created a 1,000 m buffer around each studied fragment to evaluate the degree of isolation, measured as the percentage of area covered by other fragments present within that buffer.

### Former mammalian community of the CEPE

Using past and present literature [10,32,33,40,41,42,43,44,53,54,55], we built a checklist of the medium- and large-sized mammalian species that, unless human action had caused their extirpation, could be expected to be found in the CEPE. The current conservation status of extant and known species is according to the Brazilian List of Threatened Species [56] and IUCN Red List [57].

### Line transect surveys and additional methods

Surveys of medium-sized (≤5 kg, excluding mice and marsupials) and large (>5 kg) mammals were carried out between January 2000 and January 2008 using the line transect method [58] throughout the entire year but only on sunny days (e.g., if it rained during a survey, the results were discarded). A single 1-m wide transect was arbitrarily cut in each fragment, measuring between 150 and 4,000 m in length, depending on the size and shape of the fragment as well as accessibility. Diurnal walks were carried out between 5:00 and 17:00 h at a speed of 1 km/h, with nocturnal surveys performed between 18:00 and 23:00 h, following Mendes Pontes [59]. Sample size depended on the size and shape of the fragments.

For each sighting of an individual or group, we recorded all of the standard information necessary for the calculation of abundance indexes [58,60], but due to the limited number of sightings, we could not calculate group or individual densities. We therefore used sighting rates [7,8,14]. Additionally, we estimated group size and the total kilometers walked each day. We classified the species according to their occurrence in specific sizes of forest fragments as follows: (1) rare, when species occurred in only one fragment size class; (2) occasional, when found in two or three fragment size classes; or (3) common, when found in more than three size classes. We considered a species to be extinct in a forest fragment when we did not detected it during the systematic surveys or in occasional encounters because the fragments are so small, isolated, lacking in food, simplified, and secondarized [22,29,61] that it was almost impossible that the species remained undetected in the fragment. Reinforcing our findings, no new species was added to the checklist based on spoor or dung.

### Data Analysis

We used a one-way Chi-Square Goodness of Fit statistic to test the hypothesis that the current number of species of medium-sized mammals in the CEPE is significantly lower than that registered prior to Brazilian colonization. Subsequently, we ran a Generalized Linear Model (GLZ) to analyze to what extent the vegetation type, fragment area, fragment isolation (as the percentage of forest cover in a 1,000 m buffered area), and sampling effort (as total kilometers walked), or higher-order interactions predicted the response variables (a) richness and (b) sighting rates. Explicitly considering forest type as a categorical independent variable (factor) and three continuous predictor variables as covariates (area, isolation and effort), we also employed a Linear Analysis of Covariance Model (ANCOVA). Specifically, we used a factorial ANCOVA, as we included three two-way interactions and one three-way interaction in the model [62]. The continuous covariate effort was added to the model as a weight variable only to control for response variables. We excluded the submontane evergreen and low montane semi-deciduous forest types from the GLZ analysis because of small sample size (two and one forest fragments, respectively).

Before running the models, we performed Kolmogorov-Smirnov tests to check which theoretical distribution best fit our response variables (richness and sighting rates). Richness showed a distribution that was far from a normal distribution, even after several transformation attempts, but which fitted well to a Poisson distribution (Lambda = 3.33; d = 0.297; p < 0.10). Thus, we employed a Poisson distribution with a log link-function to run this model, using Pearson’s chi-square as an overdispersion parameter estimate. Sighting rates fitted well to a log-normal distribution. Thus, the log-transformed sighting rates exhibited a normal distribution (d = 0.159; p = ns; Lilliefors p = ns). We then used a normal distribution with an identity link-function to run the GLZ for the log-transformed sighting rates. After running the models, we performed a residual analysis (raw residual normality and leverage). All GLZ analyses were carried out using IBM SPSS Statistics 19 [63].

We used the NODF nestedness metric [64] to determine whether the actual distribution of the mammalian species within the studied forest fragments exhibited a nested pattern. In a hypothetical perfect nested pattern, the set of species in any forest fragment will be a perfect subset of all of the species that precede it in a hierarchically arranged forest fragment [64]. We used a 21x21 (“fragments” x “mammal species”) presence-absence matrix and generated 1,000 random matrices to contrast the nestedness hypothesis against the null hypothesis that the distribution of the mammals among the forest fragments is stochastic. Because the NODF method is dependent on the arrangement of the matrix [64], we performed three analyses, one using a best-ordered matrix, one ordering the matrix according to the area of the fragment, and another one ordering the matrix according to the isolation of the fragment. Once we were only mainly concerned with the nestedness among fragments, rather than among species or within the entire matrix, we also explicitly reported results of the analysis using only the columns of the matrix. The NODF analysis was performed using ANINHADO software [65].

Finally, we performed a Binomial Logistic Regression [62] to predict the probability of encounter for each species according to fragment size. Because effort was not the same in all fragments we then used the kilometers walked as a weight factor controlling for the different efforts in each fragment. The data used for the above mentioned analysis are in S1 Table.

## Results

### The former and current mammalian fauna richness in the CEPE

The former mammalian community of the CEPE included 43 medium- and large-sized mammal species, whereas the current mammalian fauna comprised only 51.2% (n = 22) of those species (χ^2^ = 10.2; df = 1; p < 0.01) (Table 1), including a new dwarf porcupine, *Coendou speratus*, recently described by the first author [48], as well as those species that went extinct before description or even before being depicted by the first colonizers. Among the total number of species that appeared to be extinct in the CEPE (48.8%; n = 21) since the beginning of colonization, 41.2% (n = 7) are not referred to either in the ICMBIO list of threatened species [56] or the IUCN Red List [57], while 47% (n = 8) are included in both lists, 5.9% (n = 1) are included only in the IUCN list, and 5.9% (n = 1) only in the ICMBIO list, respectively (Table 1).

**Table 1:** Former and current mammalian fauna of northeastern Brazil in the Pernambuco Endemism Center (CEPE) and their conservation status.

Species	ICMBIO (2014)	IUCN (2015)	Mentioned by first colonizers from 16^th /^ 17^th^ century only: extinct even before depicted or seen	Mentioned / depicted by first colonizers from 16^th^ / 17^th^ century: Extinct before species confirmed	Mentioned / depicted by first colonizers from 16^th^ / 17^th^ century	Referred as occurring by scientists from 20^th^ century based on indirect evidence	Referred as occurring by scientists from 20^th^ century based on indirect evidence—Extinct before species confirmed	Referred as occurring by scientists from 21^th^ century based on indirect evidence	Referred as occurring by scientists from 21^th^ century based on indirect evidence- Extinct before species confirmed	Referred as occurring by scientists from 20^th^ and 21^th^ century based on direct evidence	Depicted by first colonizers from 16^th^ / 17^th^ century; Remained unkown to science for ~400 yrs; Re-discovered during this study	Newly described by first author (21^th^ century)
**Artiodactyla**												
**Cervidae**					** **							
*Mazama* sp. EX BK1	EX BK*1			✓	** **		✓		✓			
*Mazama* sp. EX BK2	EX BK*1			✓	** **							
**Tayassuidae**					** **							
*Tayassu pecari*	VU A2abcde+3abcde	VU A2bcde+3bcde					✓		✓			
Pecari tajacu*2					✓	✓		✓				
**Carnivora**												
**Canidae**												
Cerdocyon thous*3					✓	✓		✓				
*Speothos venaticus*	VU C1	NT		✓								
**Felidae**												
*Leopardus pardalis*	VU A4c				✓	✓		✓				
*L*. *tigrinus*	EN C1	VU A3c			✓	✓		✓				
*L*. *wiedii*	VU C1	NT		✓			✓		✓			
*Panthera onca*	A2bcd+3cd; C1	NT		✓					✓			
*Puma concolor*	VU C1			✓			✓		✓			
*P*. *yagouaroundi*	VU C1					✓		✓				
**Mustelidae**												
*Conepatus semistriatus*							✓					
Eira barbara*4					✓							
*Galictis c*.*f*. vittata*5					✓	✓		✓				
*Lontra longicaudis*		NT		✓								
**Procyonidae**												
Nasua nasua*4					✓							
*Potos flavus*							✓		✓			
*Procyon cancrivorus*					✓	✓		✓				
**Cingulata**												
**Dasypodidae**												
*Cabassous unicinctus*							✓		✓			
*Dasypus novemcinctus*					✓	✓		✓				
*D*. *septemcinctus*							✓					
*Euphractus sexcinctus*					✓	✓		✓				
Tolypeutes tricinctus*6	EN A2cd	VU A2cd		✓			✓					
**Perissodactyla**												
**Tapiridae**												
*Tapirus terrestris*		VU A2cd+3cd+4cd		✓			✓		✓			
**Pilosa**												
**Bradipodidae**												
*Bradypus variegatus*					✓	✓		✓				
**Cyclopedidae**												
*Cyclopes didactylus*				✓			✓		✓			
**Myrmecophagidae**												
*Myrmecophaga tridactyla*	VU A2d	VU A2c		✓			✓		✓			
*Tamandua tetradactyla*					✓	✓		✓				
**Primate**												
**Atelidae**												
Alouatta belzebul*7				✓			✓		✓			
*Ateles* sp. EX BK3	EX BK*1		✓									
**Callithrichidae**												
*Callithrix jacchus*					✓					✓		
**Cebidae**												
Cebus apella*8			✓				✓		✓			
*Saimiri* sp. EX BK4	EX BK*1		✓									
*Saimiri sciureus*			Accidentally introduced									
Sapajus flavius*9	A2cd; B2ab(ii,iii); C2a(i)	CE C2a(i)			✓						✓	
**Rodentia**												
**Cuniculidae**												
*Cuniculus* c.f. paca*3,*10					✓							
**Dasyproctidae**												
*Dasyprocta prymnolopha*					✓	✓		✓				
**Erethizontidae**												
Coendou prehensilis*11					✓	✓		✓				
*Coendou speratus*	EN B1ab(iii)											✓
**Hydrochaeridae**												
*Hydrochoerus hydrochaeris*					✓	✓		✓				
**Sciuridae**												
*Guerlinguetus alphonsei*					✓	✓		✓				
**Lagomorpha**												
*Sylvilagus brasiliensis*					✓	✓		✓				

*1: Extinct Before Known; We propose this category to distinguish it from the other IUCN categories and criteria, which do not include species that went extinct before known or scientifically described

*2: Last time seen in 2006. A new effort to find them and collect hair samples for genetic studies failed

*3: Two morphotypes occur: the '*cat-faced*' and the '*dog-faced*' crab-eating fox. Urgent molecular studies needed

*4: Currently common widespread despite not listed to the region in the key publications. As a result of this study we extended its distributional range to the CEPE

*5: During this study we saw, and also found a dead specimen in a burnt sugar-cane plantation, and concluded that the species is not *G. vittata*. Until urgent molecular studies are carried out, we assume it is *G*. c.f. *cuja*

*6: Until recently this species was considered endemic to the dry-scrub caatinga forests of northeastern Brazil; During this long-term study we found out that the species once extended its distributional range into the CEPE and that it had been extinct before present-day scientists knew they occurred there.

*7: Vocalizations assumedly from this species has been heard by local primatology student, but subsequent efforts to locate the group did not succeed

*8: Since this species occurs in the contiguous dry-scrub caatinga forests, it may have been sympatric with *Sapajus flavius* in the CEPE

*9: Single group discovered isolated for over 30 years in the smallest and last fragment studied (4 ha) [83]

*10: From the Amazonia to the Atlantic forest the genus *Cuniculus* has the same two morphotypes, the '*deep chin*', '*pitbull*' or '*ladle paca*', which is larger and has much deeper and wider zygomatic arch, and the 'commom', which has it much less conspicuous; Preliminary molecular analysis has shown that in the CEPE it is a different, therefore new species; Description in progress.

*11: This is the correct species referred and depicted by the first colonizers from the type locality Pernambuco State in the 17th century, upon which Linnaeus (1758) based his description; This species is endemic to the CEPE and other species described outside this distributional range needs revision [84]

**Fig 2:**
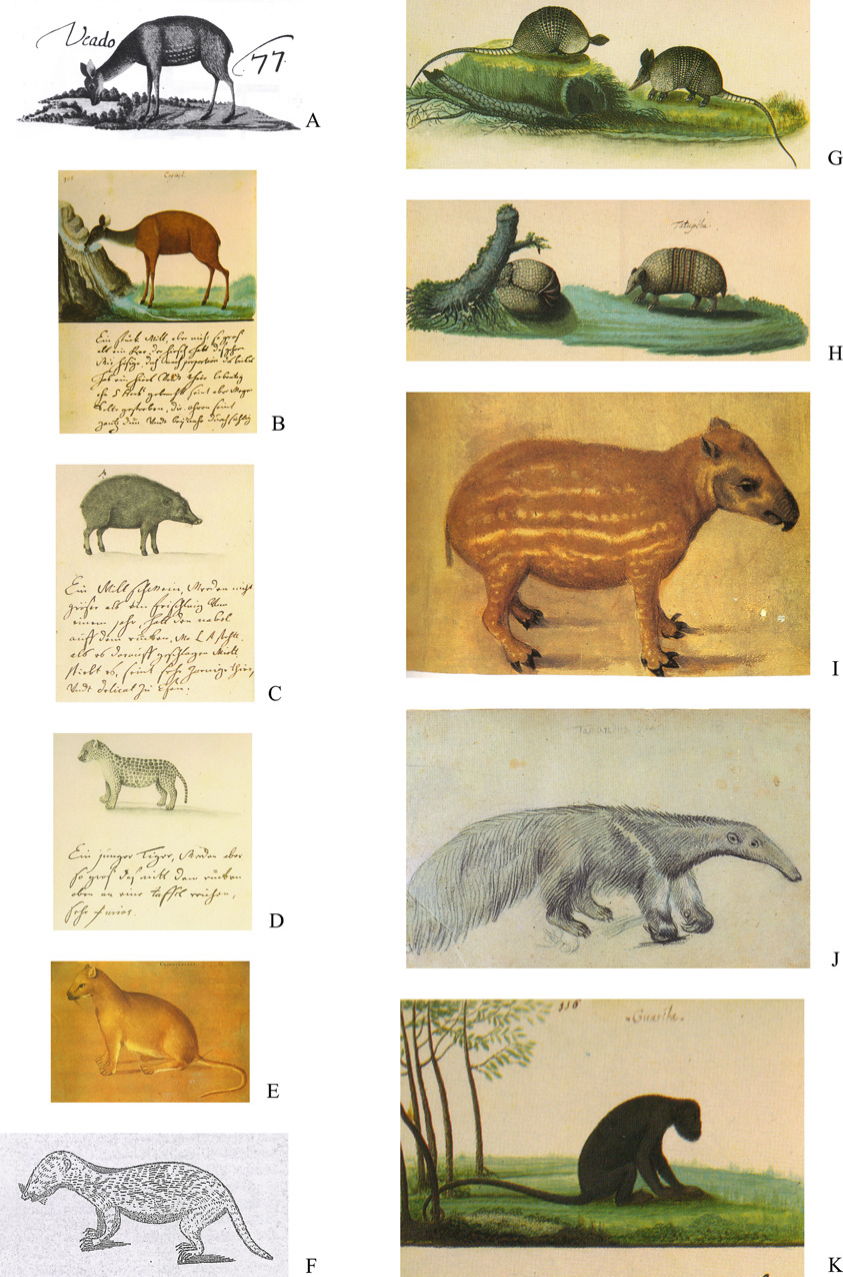
Extinct large mammal species depicted by the first colonizers of the Atlantic coast of northeastern Brazil. A and B) c.f. *Mazama americana*; C) *Tayassu pecari*; D) *Panthera onca*; E) *Puma concolor*; F) *Lutra longicaudis*; G) Possibly *Cabassous unicinctus* (Emmons and Feer, 1997: 10–13 movable bands; tail narrow and long; ears large, funnel-like), but about which Marcgrave says: “This is armoured animals and are able to pull the head and paws, doubling themselves into a ball". Since the only armadillos capable of rolling into a ball are those of the genus *Tolypeutes* (Eisenberg and Redford, 1999), we assume that Marcgrave was not sure about the distinction between *T*. *tricinctus* and other armadillo species; H) *Tolypeutes tricinctus*; I) *Tapirus terrestris*; J) *Myrmechophaga tridactyla*; K) *Alouatta belzebul*.

Some of these species have been referred to the CEPE in the old literature and paintings of the first colonizers in the 16^th^ and 17^th^ century [32,33,40,41,42,43,44], but not by contemporary scientists [53,54,55,56,57], despite the fact that they are currently widespread in the region (e.g. *Cuniculus paca*) [10] (Fig 2A–2K) (Table 1).

Some other species had not been referred to the CEPE until the turn of the 21^th^ century, such as *Eira barbara*, *Nasua nasua*, and *Speothos venaticus* [66,67], although in the case of the latter species, it was already regionally extinct and threatened of extinction in the remainder of their range [56,57] when recognized as occurring in the region (Fig 2A–2K) (Table 1).

All the large-sized mammals are currently extinct in the CEPE, namely white-lipped peccary (*Tayassu pecari*), jaguar (*Panthera onca*), puma (*Puma concolor*), Brazilian tapir (*Tapirus terrestris*), and giant ant-eater (*Myrmecophaga tridactyla*) (Table 1). They were documented and depicted by the first colonizers when they arrived in the Atlantic coast of northeastern Brazil and inferred to occur in the CEPE by contemporary scientists, but went extinct before they could be registered or any specimens collected [10] (Fig 2A–2K). In the case of the deer (*Mazama* spp.) their occurrence was never documented by contemporary scientists; they went extinct without being registered.

Gandavo, in 1575 [32], and Salvador, in 1627 [33] also referred to a yellow-olive squirrel monkey (probably a *Saimiri* sp.), and in 1647, Barlaeus [42] referred to a rufous long-tailed spider monkey (probably a *Ateles* sp.) as also occurring in the CEPE, but no illustrations or any subsequent records of these species were found for the region. The newly described dwarf porcupine (*Coendou speratus)*, has already been classified as endangered by the Brazilian authorities [56] due to its very restricted distribution, and highly fragmented and declining population, although it has still not been evaluated by IUCN [57]. Current records of a squirrel monkey (*Saimiri* sp.), from the Saltinho Biological Reserve, are of an introduced Amazonian species, individuals of which were confiscated by the National Environment Office (IBAMA) and accidentally released in the reserve some 30 years ago (Table 1).

No individual fragment hosted the entire remaining mammal community. In the largest fragment, the Coimbra forest, we found the largest number of species (95.2%, n = 20). We observed 14 species in the medium-sized fragments, (66.7%), 9 species (42.8%) in the small fragments, and only 4 (19%) in the very small fragments. The common marmoset (*Callithrix jacchus*) and the common squirrel (*Guerlinguetus alphonsei*) were found in all fragment size classes. The common marmoset and 10 other species (52.4% in total) were also found in the open surrounding matrix (Table 2).

**Table 2:** Occurrence of the medium- and large-sized mammals in the different size classes of the studied forest fragments in northeastern Brazil.

Species	≤ 10 (ha)	10.1–100 (ha)	100.1–1,000 (ha)	3,478.3 (ha)	Surrounding non-forested matrix
*Bradypus variegatus*	**X**	**X**		**X**	
*Callithrix jacchus*	**X**	**X**	**X**	**X**	**X**
*Cerdocyon thous*		**X**	**X**	**X**	**X**
*Coendou prehensilis*		**X**	**X**	**X**	
*Cuniculus paca*				**X**	
*Dasyprocta prymnolopha*		**X**	**X**	**X**	**X**
*Dasypus novemcinctus*			**X**	**X**	
*Eira barbara*			**X**	**X**	**X**
*Euphractus sexcinctus*			**X**	**X**	
*Galictis vittata*				**X**	**X**
*Guerlinguetus alphonsei*	**X**	**X**	**X**	**X**	
*Hydrochoerus hydrochaeris*			**X**	**X**	**X**
*Leopardus pardalis*				**X**	
*L*. *tigrinus*				**X**	
*Nasua nasua*		**X**	**X**	**X**	**X**
*Pecari tajacu*			**X**	**X**	
*Procyon cancrivorous*			**X**	**X**	**X**
*Puma yaguarondi*				**X**	**X**
*Sapajus flavius*	**X**	**X**			**X**
*Sylvilagus brasiliensis*			**X**	**X**	**X**
*Tamandua tetradactyla*			**X**	**X**	
**Total:**	**4**	**9**	**14**	**20**	**11**

Out of 21 species present in the studied fragments (excluding the non-forested matrix), 5 species (23.8%) were rare, occurring in only the single largest forest fragment; 14 species (66.7%) were occasional, occurring in two or three fragment size classes; and only two species (9.5%) were common and were found in all four fragment size classes. Thus, the data suggest that 73.3% of the remaining forest fragments of the CEPE (very small fragments, with a mean size of 2.8 ha) host only four species of medium-sized mammals: *Callithrix jacchus*, *Guerlinguetus alphonsei*, the brown-throated three-toed sloth (*Bradypus variegatus*) and the blond capuchin (*Sapajus flavius*) (Table 3). In 24% of the remaining fragments (small fragments, with a mean size of 27 ha), nine species were registered; in 2.5% (medium-sized fragments, with a mean size of 241 ha), 14 species were registered; and in only 0.2% (large fragments, with a mean size of c.a. 2,100 ha), 20 species were registered (Table 3).

**Table 3:** Remaining forest fragments of the Pernambuco Endemism Center (CEPE) and their medium- and large-sized mammalian richness.

Size classes	Total no. of	Total no. of	Total area	Mean size (± SD)
	mammal species	fragments	in the CEPE (ha)	
**≤10**	4	13,619	38,503.96	2.83 (2.24)
**10.1–100**	9	4,460	121,081.24	27.15 (19.21)
**100.1–1,000**	14	474	114,440.33	241.43 (169.85)
**>1,000**	20	23	48,347.33	2,102.05 (1,505.75)
**TOTAL:**	**21**	**18,576**	**322,372.86**	

### Abundance of the current mammalian fauna in the CEPE

Independent of the number or type of forest fragments, the most abundant species was the common marmoset (*Callithrix jaccchus*), which was sighted at a rate of 74.6 individuals/10 km walked. The tayra (*Eira barbara*) was the next most abundant, with 12 ind/10 km, followed by the squirrel *Guerlinguetus alphonsei*, with 6.6 ind/10 km. The least abundant species were the crab-eating raccoon (*Procyon cancrivorous*), the jaguarondi (*Puma yagouaroundi*) and the common rabbit (*Sylvilagus brasiliensis*), which were observed only occasionally and always outside systematic surveys, suggesting sighting rates below a detectable level (Table 4).

**Table 4:** Abundance of the medium- and large-sized mammals in the Pernambuco Endemism Centre, Brazil.

Forest type /	Area (ha) of	Transect	Degree of	ha(%)	∑	∑	Species^a^	Sightings/	Mean	Sighting
fragment	fragment	(meters)	isolation	surveyed	Sightings	Species		species	group size	rate
**SUBMONTANE EVERGREEN**										
Coimbra	3 478.3	4,000	6.92	20 (0.6%)	28	20	*Agouti paca*	1	1	0.03
(08°59'42"W; 35°50'27‴S)							*Bradypus variegatus*	0	0	0
							*Callithrix jacchus*	0	0	0
							*Cerdocyon thous*	0	0	0
							*Coendou prehensilis*	0	0	0
							*Dasyprocta primnolopha*	10	1	0.3
							*Dasypus novemcinctus*	0	0	0
							*Eira barbara*	1	1	0.03
							*Euphractus sexcinctus*	3	1	0.04
							*Galictis vittata*	1	1	0.03
							*Guerlinguetus alphonsei*	4	1.2	0.12
							*Hydrochaerus hydrochaeris*	0	0	0
							*Leopardus pardalis*	1	1	0.01
							*Leopardus tigrinus*	1	1	0.01
							*Nasua nasua*	5	4.7	0.15
							*Pecari tajacu*	1	6	0.03
							*Procyon cancrivorus*	0	0	0
							*Puma yaguarondi*	0	0	0
							*Sylvilagus brasiliensis*	0	0	0
							*Tamandua tetradactyla*	0	0	0
										**0.75**
Aquidabã	24	830	0.89	4.1 (17.1%)	1	1	*Callithrix jacchus*	1	1	0.9
(08°58'53"S; 35°54'25"W)										**0.9**
**LOWLAND EVERGREEN**										
Saltinho	548	3,175	1.87	31.7 (5.7%)	74	7	*Callithrix jacchus*	53	4.2	6.8
(08°45'00"S; 35°10'00"W)							*Dasyprocta prymnolopha*	11	1	1.41
							*Dasypus novemcinctus*	1	1	0.08
							*Eira barbara*	1	1	0.13
							*Nasua nasua*	5	2.6	0.64
							*Saimiri sciureus*	1	20	0.13
							*Tamandua tetradactyla*	2	1.5	0.16
										**9.35**
Tapacurá	382	2,000	1.67	20 (5.2%)	115	2	*Callithrix jacchus*	115	2.4	11.5
(08°03'00"S; 34°55'00"W)							*Dasyprocta prymnolopha*	0	0	0
										**11.5**
Cuxiu	118	900	8	2.2 (1.8%)	4	1	*Callithrix jacchus*	4	1.75	4
(08°13'38"S; 35°03'45"W)										**4**
Charles Darwin	60	1,500	15.76	4.5 (7.5%)	5	5	*Callithrix jacchus*	4	3.25	1.95
(07°48'56"S; 34°57'11"W)							*Bradypus variegatus*	0	0	0
							*Cerdocyon thous*	0	0	0
							*Dasyprocta prymnolopha*	0	0	0
							*Guerlinguetus alphonsei*	1	1	0.5
										**2.45**
São Braz	37	700	10.52	4.3 (11.6%)	11	2	*Callithrix jacchus*	10	2.1	10
(08°13'28"S; 35°04'03"W)							*Guerlinguetus alphonsei*	1	1	1
										**11**
Gengibre	19.8	250	2.12	2.5 (12.6%)	2	2	*Sapajus flavius*	1	12	1
(08°31'33"S; 35°03'16"W)							*Eira barbara*	1	1	1
										**2**
Mingú	13	250	6.39	2.5 (18.6%)	2	2	*Sapajus flavius*	1	18	1
(08°31'02"S; 35°03'15"W)							*Nasua nasua*	1	1	1
										**2**
Xangô	9	500	13.8	2.5 (28%)	13	2	*Bradypus variegatus*	2	1	2
(08°14'23"S; 35°03'56"W)							*Callithrix jacchus*	11	2.2	11
										**13**
Vale das Aguas	8	350	56.75	0.8 (10%)	3	2	*Callithrix jacchus*	2	3.5	4
(7°54'49"S; 35°3'25,4"W)							*Guerlinguetus alphonsei*	1	1	2
										**6**
Café	7	450	13.94	2.2 (32.3%)	9	2	*Bradypus variegatus*	2	1	1
(08°14'12"S; 35°02'60"W)							*Callithrix jacchus*	7	2.1	7
										**8**
Bulandí	4	150	4.44	1.5 (37.5%)	4	4	*Sapajus flavius*	3	22.3	4.2
(08°32'10"S; 35°02'50"W)							*Nasua nasua*	1	1	1.4
							*Callithrix jacchus*	0	0	0
							*Agouti paca*	0	0	0
										**5.6**
**SUBMONTANE SEMI-DECIDUOUS**										
Quengo	500	1,900	5.94	3.2 (0.6%)	3	3	*Callithrix jacchus*	1	2	1.25
(08°43'04"S; 35°50'27"W)							*Dasyprocta prymnolopha*	0	0	0
							*Nasua nasua*	2	1	2.5
										**3.75**
Fervedouro	300	1,000	2.58	0.6 (0.2%)	2	2	*Callithrix jacchus*	2	2	2.86
(08°45'08"S; 35°51'36"W)							*Guerlinguetus alphonsei*	0	0	0
										**2.86**
Cachoeira	271	3,000	12.89	15 (5.5%)	14	9	*Callithrix jacchus*	0	0	0
(08°56'44"S; 36°03'36"W)							*Eira barbara*	1	1	0.06
							*Euphractus sexcinctus*	2	1	0.07
							*Hydrochaerus hydrochaeris*	1	3	0.04
							*Nasua nasua*	7	4	0.41
							*Pecari tajacu*	1	3	0.06
							*Procyon cancrivorus*	0	0	0
							*Guerlinguetus alphonsei*	2	1	0.12
							*Sylvilagus brasiliensis*	0	0	0
										**0.75**
Capoeirão	122	1,500	15.12	7.5 (6.1%)	9	8	*Cerdocyon thous*	1	1	0.04
(08°55'1"S; 36°04'17"W)							*Coendou prehensilis*	1	1	0.1
							*Euphractus sexcinctus*	1	1	0.04
							*Nasua nasua*	4	4.5	0.25
							*Guerlinguetus alphonsei*	1	1	0.06
							*Pecari tajacu*	1	5	0.06
							*Procyon cancrivorus*	0	0	0
							*Callithrix jacchus*	0	0	0
										**0.55**
Ageró	50	500	6.4	2.8 (5.6%)	4	3	*Callithrix jacchus*	4	1.6	5.33
(08°44'16"S; 35°50'33"W)							*Coendou prehensilis*	0	0	0
							*Dasyprocta prymnolopha*	0	0	0
										**5.33**
Espelho	50	1,000	19.58	5.6 (11.2%)	2	2	*Guerlinguetus alphonsei*	2	1	2.9
(08°43'12"S; 35°50'40"W)							*Callithrix jacchus*	0	0	0
										**2.9**
Bom Jesus	41	500	24.3	2.5 (6%)	6	2	*Callithrix jacchus*	2	2.5	2.9
(09°01'16"S; 36°10'32"W)							*Nasua nasua*	4	1	5.7
										**8.6**
**LOW MONTANE SEMI-DECIDUOUS**										
Brejo dos Cavalos	354	2,400	3.23	24 (6.7%)	23	2	*Callithrix jacchus*	22	3.3	5.1
(08°22'48"S; 36°02'24"W)							*Eira barbara*	1	1	0.23
										**5.33**

^a^Including species that were sighted outside of systematic surveys in the surrounding matrix

### Environmental determinants of the richness and abundance of the mammalian fauna in the forest fragments of the CEPE

The Linear Analysis of Covariance (ANCOVA) showed that neither the species richness nor the sighting rate is controlled by vegetation type, fragment area, fragment isolation, or any interaction among these factors (Table 5).

**Table 5:** Whole models (comparing the fitted model against the intercept-only model) and model effect decompositions for each of the two response factors (species richness and sighting rates) according to the Generalized Linear Model analysis (Factorial Analysis of Covariance—ANCOVA).

*Model*	*Distribution (link function)*	*Chi-Square*	*DF*	*p*
***Species richness***	*** ***			
Whole model*	Poisson** ***(Log)***	***20*.*71***	***8***	***0*.*008***
***Intercept***	*** ***	***3*.*92***	***1***	***0*.*048***
***Vegetation types***	*** ***	***0*.*476***	***1***	***0*.*490***
***Area***	*** ***	***0*.*398***	***1***	***0*.*528***
***Isolation***	*** ***	***0*.*494***	***1***	***0*.*482***
***Effort***	*** ***	***1*.*279***	***1***	***0*.*258***
Vegetation*Area	*** ***	***0*.*348***	***1***	***0*.*555***
Vegetation*Isolation	*** ***	***0*.*395***	***1***	***0*.*530***
Area*Isolation	*** ***	***1*.*049***	***1***	***0*.*306***
Vegetation*Area*Isolation	*** ***	***0*.*03***	***1***	***0*.*960***
	*** ***			
***Sighting rates (log)***	*** ***			
***Whole model***	***Normal (Identity)***	***16*.*942***	***8***	***0*.*031***
***Intercept***	*** ***	***15*.*396***	***1***	***<0*.*001***
***Vegetation types***	*** ***	***0*.*042***	***1***	***0*.*839***
***Area***	*** ***	***0*.*676***	***1***	***0*.*411***
***Isolation***	*** ***	***0*.*107***	***1***	***0*.*744***
***Effort***	*** ***	***2*.*969***	***1***	***0*.*085***
Vegetation*Area	*** ***	***0*.*799***	***1***	***0*.*371***
Vegetation*Isolation	*** ***	***0*.*003***	***1***	***0*.*954***
Area*Isolation	*** ***	***0*.*535***	***1***	***0*.*464***
Vegetation*Area*Isolation	*** ***	***0*.*764***	***1***	***0*.*382***

Significant values are presented in bold.

*Overdispersion parameter estimated by Pearson’s chi-square

** Over-dispersion = 0.787

The NODF metric revealed that the medium- and large-sized mammal assemblages were significantly nested, independent of the prior matrix ordering (Table 6). This finding indicated that the degree of nestedness of the mammals among fragments was higher than the degree of nestedness obtained from 1,000 random samples, even when we arranged the presence/absence according to the best-ordered matrix, area, or isolation. However, when taking into consideration only the fragments in the matrix (our main aim) and using a null model based on randomness assigned within the rows, the NODF analysis showed that the mammal assemblages are not more nested than the random matrices (Table 7). That is to say, nestedness is not related to the area of the fragments.

**Table 6:** Measure of nestedness for the medium- and large-sized mammal assemblages in the 21 forest fragments studied according to the arrangement of three matrices.

Whole matrix	N_Total	NODF(Er)	P(Er)	NODF(Ce)	P(Ce)
**Best-ordered matrix**	58.35	20.49	<0.001	29.96	<0.001
**Area-ordered matrix**	29.11	10.08	<0.001	14.18	<0.001
**Isolation-ordered matrix**	34.68	10.65	<0.001	16.8	<0.001

N_total: total matrix nestedness; NODF(Er): nestedness of the null model in which presences are randomly assigned to any cell within the matrix; P(Er): significance of NODF based on this null model; NODF(Ce): nestedness of the null model in which the probability of a cell, aij, showing a presence is (Pi/C þPj/R)/2, where Pi represent the number of presences in row i, Pj the number of presences in column j, C the number of columns, and R the number of rows; P(Ce): the significance of NODF based on this null model.

**Table 7:** Measure of column-only nestedness for the medium- and large-sized mammal assemblages in the 21 forest fragments studied according to the arrangement of three matrices.

Column-only analysis	N_Columns	P(col)	P(Li)
**Best-ordered matrix**	52.53	<0,001	0.004
**Area-ordered matrix**	15.63	<0,001	0.82
**Isolation-ordered matrix**	25.96	<0,001	0.086

N_columns: total column nestedness; P(Col): significance of NODF assuming that the presences are randomly assigned within the columns; P(Li): significance of NODF assuming that the presences are randomly assigned within the rows.

**Fig 3:**
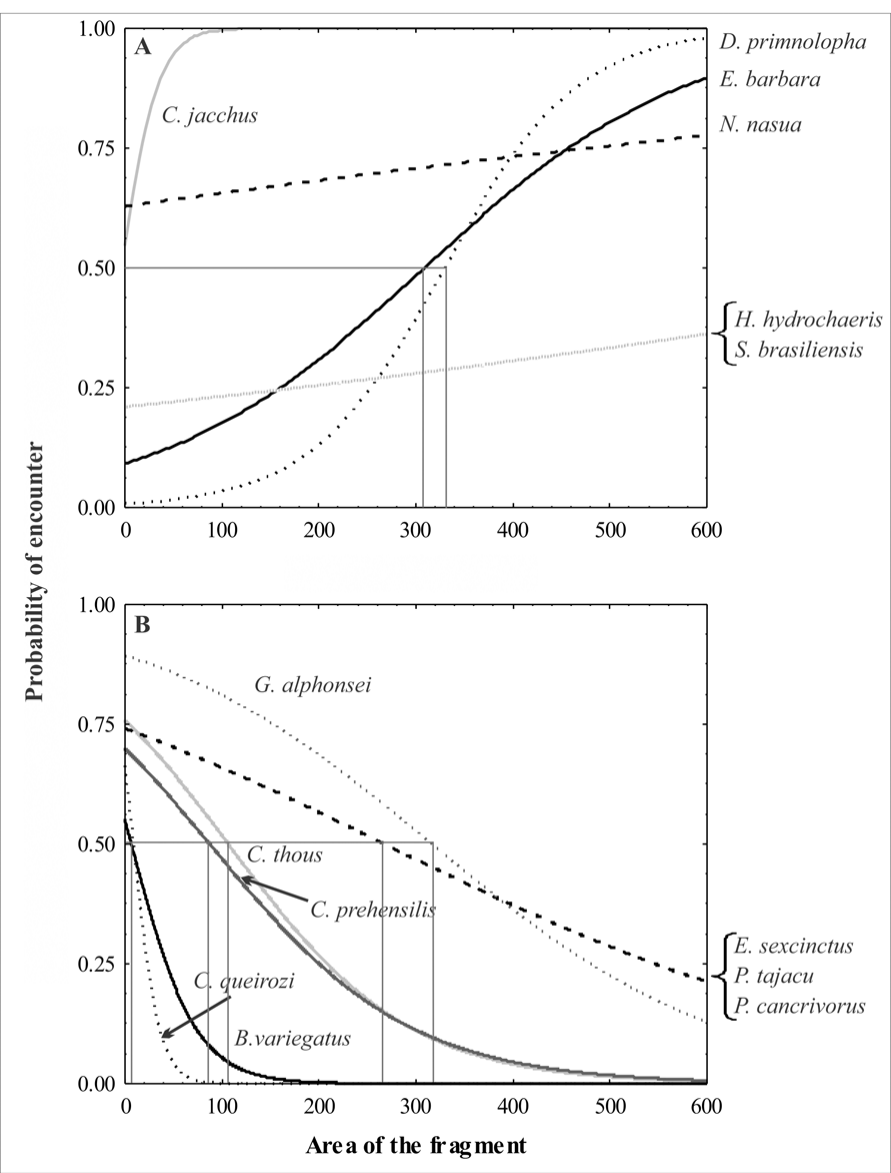
**Positive (a) and negative (b) effects of the area of the fragments on the probability of encountering medium-sized mammal species in the CEPE, according to a Binomial Logistic Regression**.

The probability of encounter for the studied species varied widely. According to a binomial logistic analysis, *Callithrix jacchus* and *Nasua nasua* showed increased chances of being detected with increased fragment size, and in all fragment sizes, these species had >50% chance of being detected; *Hydrochaerus hydrochaeris* and *Sylvilagus brasiliensis* also exhibited an increased chance of being detected with increased fragment size, though their chance of detection was <50% in all cases. *Dasyprocta prymnolopha* and *Eira barbara* presented increased chances of detection with increased fragment size, but were more likely to be found (>50% chance) in fragments larger than 300 ha. In contrast, *Sapajus flavius* and *Bradypus variegatus*, in addition to showing a reduced chance of being detected with increased fragment size, had detection probabilities of <50% in all cases. *Cerdocyon thous* and *Coendou prehensilis* also showed a reduced chance of being detected with increased fragment size and were more likely to be found (>50% chance) in fragments smaller than 100 ha; the same trend was found for *Euphractus sexcinctus*, *Pecari tajacu*, *Procyon cancrivorous*, and *Guerlinguetus alphonsei*, though these species were more likely to be found in fragments smaller than 300 ha (Table 8; Fig 3A and 3B). The other recorded species did not have any significant trend.

**Table 8:** Whole models (comparing the fitted model against the intercept-only model) and the effect of the fragment area on the probability of medium-sized mammal species encounters according to the Binomial Logistic Regression.

Species	Estimate	SE	χ^2^	P-value
***Agouti paca***				
Whole Model			78.9665	**< .0001**
Intercept	72.1088	18441.038	0.00	0.9969
Area	-13.0884	3027.1779	0.00	0.9969
***Bradypus variegatus***				
Whole Model			131.94	**< .0001**
Intercept	0.2086	0.2800	0.56	0.4563
Area	-0.0307	0.0046	44.52	**< .0001**
***Callithrix jacchus***				
Whole Model			87.7275	**< .0001**
Intercept	0.1910	0.3652	0.27	0.6009
Area	0.0537	0.0133	16.31	**< .0001**
***Sapajus flavius***				
Whole Model			134.5865	**< .0001**
Intercept	0.6857	0.3845	3.18	0.0745
Area	-0.0763	0.0175	18.91	**< .0001**
***Cerdocyon thous***				
Whole Model			205.7778	**< .0001**
Intercept	1.1475	0.1824	39.57	**< .0001**
Area	-0.0107753	0.0009786	121.23	**< .0001**
***Coendou prehensilis***				
Whole Model			172.0976	**< .0001**
Intercept	0.8495	0.1766	23.14	**< .0001**
Area	-0.0097	0.0009	107.21	**< .0001**
***Dasyprocta primnolopha***				
Whole Model			362.0748	**< .0001**
Intercept	-4.7913973	0.3704	167.33	**< .0001**
Area	0.0145	0.0012	152.38	**< .0001**
***Dasypus novemcinctus***				
Whole Model			474.3666	**< .0001**
Intercept	-452.7074	101082.37	0.00	0.9964
Area	0.8660	192.3983	0.00	0.9964
***Eira barbara***				
Whole Model			170.9121	**< .0001**
Intercept	-2.2855	0.19624	135.64	**< .0001**
Area	0.0074	0.0007	126.07	**< .0001**
***Euphractus sexcinctus***				
Whole Model			60.0989	**< .0001**
Intercept	1.0538	0.1547	46.39	**< .0001**
Area	-0.0039	0.0005	54.28	**< .0001**
***Guerlinguetus alphonsei***				
Whole Model			147.4559	**< .0001**
Intercept	2.1270	0.1888	126.85	**< .0001**
Area	-0.0067	0.0006	114.17	**< .0001**
***Hydrochaerus hydrochaeris***				
Whole Model			5.4490	**0.0196**
Intercept	-1.3226	0.1672	62.55	**< .0001**
Area	0.0013	0.0006	5.46	**0.0195**
***Nasua nasua***				
Whole Model			5.0445	**0.0247**
Intercept	0.5271	0.1520	12.03	**0.0005**
Area	0.0012	0.0005	4.94	**0.0262**
***Pecari tajacu***				
Whole Model			60.0989	**< .0001**
Intercept	1.0538	0.1547	46.39	**< .0001**
Area	-0.0039	0.0005	54.28	**< .0001**
***Procyon cancrivorus***				
Whole Model			60.0989	**< .0001**
Intercept	1.0538	0.1547	46.39	**< .0001**
Area	-0.0039	0.0005	54.28	**< .0001**
***Sylvilagus brasiliensis***				
Whole Model			5.4489	**0.0196**
Intercept	-1.3226	0.1672	62.55	**< .0001**
Area	0.0013	0.0005	5.46	**0.0195**
***Tamandua tetradactyla***				
Whole Model			474.3666	**< .0001**
Intercept	-452.7074	101082.37	0.00	0.9964
Area	0.8660	192.3982	0.00	0.9964

Significant values are presented in bold.

## Discussion

After more than 500 years of colonization, the Atlantic forest of northeastern Brazil, specifically the CEPE, has lost at least half of its medium-sized and all large-sized mammals. The richness and abundance of the remaining mammals are not predicted by the fragment area, fragment isolation, vegetation type or any interaction among these factors. In addition, neither fragment area nor fragment isolation accounts for the detected nestedness pattern. For the within-group analyses, some species did not occur as would be expected according to the variation in the fragment area.

During our surveys in forest fragments of the remaining 5.6% of the CEPE, more than 500 years after the beginning of the colonization process, we still observed intense human interference, especially hunting, with or without feral dogs [68], which have led to the extinction of all large mammals of this hotspot. Despite the fact that deer (*Mazama* spp.), white-lipped peccary (*Tayassu pecari*), jaguar (*Panthera onca*), puma, *Puma concolor*, Brazilian tapir (*Tapirus terrestris*), and giant anteater (*Myrmecophaga tridactyla*) have been reported and/or depicted by the first colonizers [32,33,40,41,42, 43,44], and even referenced in current literature [53,54,55,56,57], they have never been seen in the wild or collected by contemporary scientists working in the CEPE.

Considering that white-lipped peccaries, jaguars, Brazilian tapirs, and giant anteaters are confirmedly extinct in both CEPE (this study) and CEBA [49], this indicates a total decrease of almost 90.000 km^2^ in the former geographic range of these extinct large mammals and highlights the critical importance of regional extinctions for effective species conservation.

The populations of other species, such as medium-sized cats (e.g., jaguarondi, *Puma yaguarondi*, ocelot, *Leopardus pardalis*, oncilla, *Leopardus tigrinus*), the much-hunted river otter (*Lontra longicaudis*), the collared peccary (*Pecari tajacu*) and the critically endangered blonde capuchin (*Sapajus flavius*) are not sustainable in the long term. They are “living dead” species, which will most probably become extinct in the next decades [69,70,71] unless very effective conservation measures are implemented. Some of these species are not referred to in the global, national or international lists of threatened species, and at the same time, some of these lists are not congruent with each other, which can greatly impede their effective use as a conservation tool.

Extinctions of species that used to live in small forest patches, soon after they are described, is already expected, such as happened with the Pernambuco pigmy-own (*Glaucidium mooreorum*) in the same forest fragments as in this study [39]. This should be the case of the recently described dwarf porcupine (*Coendou speratus*), still not evaluated by the IUCN [57] and the recently redescribed blond capuchin (*Sapajus flavius*), which live in isolated and small forest patches. But besides the expected extinctions, the current profound changes in species composition may further harm other species. This could be the case of the two most abundant species, the common marmoset (*Callithrix jacchus*) and the tayra (*Eira barbara*), which can become powerful predators.

The two unprecedented references to the Amazonian primates *Saimiri* sp. and *Ateles* sp. as occurring in the CEPE [32,33] provide remarkable evidence that a number of species may have gone extinct in this hotspot´s hotspot before contemporary scientists could describe them. This could be the case of the deer (*Mazama* spp.) of the region, which were referenced and depicted by the first colonizers, but went extinct before they could be described, as there are no contemporary reports of these species in the CEPE [72]. Similarly, Lees and Pimm [39] have shown that this is also the case of some species of birds, which the first colonizers depicted for the northeastern Atlantic forest, and that went extinct before they could be described (e.g. a curassow, *Crax*, with a yellow beak), their paintings being the only record available.

The remaining medium-sized mammal community is highly simplified and homogenized in comparison to the previous mastofauna described in the literature [14,40,44,53,54,55]. The same is true for small mammals [3] and trees [29,61,73]. We hypothesize that if the ongoing reduction in size and loss of existing fragments continue in the same rate the 21^st^ century medium-sized mammalian fauna of this region (73.3% of which is formed by forest fragments with a mean size of 2.8 ha (n = 13,619)) should comprise only four species: *Callithrix jacchus*, *Guerlinguetus alphonsei*, *Bradypus variegatus* and *Sapajus flavius*.

We considered some species to be extinct from a forest fragment when we did not detected them during the systematic surveys, occasional encounters or based on spoor or dung because despite the limitations inherent to the inference that a species may be extinct [74], the fragments are so small (see Table 3), isolated, lacking in food, simplified, and secondarized [29,61,73] that it is almost impossible that these species would remain undetected in the area. Our findings are also supported by Canale et al. [61] who came to the same conclusion in the nearby CEBA using only interviews with locals.

Melo et al. [68] found that hunting pressure, determined based on gun shots heard, the presence of humans, hunting trails, hunting platforms, and the presence of feral dogs, was positively related to species diversity and sighting rates. This suggests that higher richness and abundance resulted in higher hunting pressure, contradicting previous findings [75, 76]. Although the mammal distribution conforms to the principle of nested subsets [77], this nestedness was not related to fragment size or isolation, as would be expected [7, 31]. The existence of these fragments depends on the landowner and the local people who exploit them unsustainably and arbitrarily. After 500 years of systematic destruction, the northeastern Atlantic forest of Brazil is in a post-nestedness state in regard to fragment area, since any species can be found in any size of forest fragment.

The remaining living-dead species did not conform to the expected pattern in which the probability of occurrence increases with increasing patch area [78,79,80], and larger species with lower abundances are at higher risk of extinction [81,82]. Their occurrence varied unpredictably, with larger species even showing a decreased probability of detection with increasing patch size. This is most likely determined by their ability to adapt to a novel simplified diet, to efficiently use the surrounding matrix without being engulfed by the sink effect [83,84], and to escaping rampant hunting [68].

The 21^st^ century medium-sized mammalian fauna in this hotspot comprises only three or four species that can survive in very small fragments and that do not depend on crossing the open matrix to maintain viable populations. These are the species that have survived generations of “relaxation” [2]. The population sink effect [15] also plays an important role in the CEPE, since approximately 50% of the recorded mammals were observed in the open matrix.

Finally, the mammalian community of the CEPE has suffered a mass extinction of all large and some medium-sized species, and even the medium-sized remaining community is small and includes below minimum viable populations [69]. These populations are genetically isolated, subjected to the sink effect, unpredictable from an ecological perspective and consist mostly of “living dead” species [15]. The species that remain are unpredictably distributed in the different sizes of forest fragments, and their future appears to be highly uncertain. Therefore, the biota of the CEPE may represent a typical example of what will happen to preserved or less severely fragmented forests worldwide in the near future, where very strict conservation measures will have to be taken into account to ensure the long-term maintenance of wildlife populations.

## Supporting Information

S1 Table Vegetation type, fragment name and area, sighting rate, richness, effort, fragment isolation, and presence / absence matrix of the 21 studied medium-sized mammals distributed in 21 forest fragments in the Pernanmbuco Endemism Center, Atlantic forest of northeastern Brazil. Ordered according to fragment size.
(XLS)
